# ERM Proteins at the Crossroad of Leukocyte Polarization, Migration and Intercellular Adhesion

**DOI:** 10.3390/ijms21041502

**Published:** 2020-02-22

**Authors:** Almudena García-Ortiz, Juan Manuel Serrador

**Affiliations:** Interactions with the Environment Program, Immune System Development and Function Unit, Centro de Biología Molecular “Severo Ochoa” (CBMSO). CSIC-UAM, 28049 Madrid, Spain; almudenagor@gmail.com

**Keywords:** ezrin, moesin, actin, leukocytes, polarization, immune synapse

## Abstract

Ezrin, radixin and moesin proteins (ERMs) are plasma membrane (PM) organizers that link the actin cytoskeleton to the cytoplasmic tail of transmembrane proteins, many of which are adhesion receptors, in order to regulate the formation of F-actin-based structures (e.g., microspikes and microvilli). ERMs also effect transmission of signals from the PM into the cell, an action mainly exerted through the compartmentalized activation of the small Rho GTPases Rho, Rac and Cdc42. Ezrin and moesin are the ERMs more highly expressed in leukocytes, and although they do not always share functions, both are mainly regulated through phosphatidylinositol 4,5-bisphosphate (PIP_2_) binding to the N-terminal band 4.1 protein-ERM (FERM) domain and phosphorylation of a conserved Thr in the C-terminal ERM association domain (C-ERMAD), exerting their functions through a wide assortment of mechanisms. In this review we will discuss some of these mechanisms, focusing on how they regulate polarization and migration in leukocytes, and formation of actin-based cellular structures like the phagocytic cup-endosome and the immune synapse in macrophages/neutrophils and lymphocytes, respectively, which represent essential aspects of the effector immune response.

## 1. Introduction

The plasma membrane (PM)-associated cytoskeleton, namely the cell cortex, is a dense network of microfilaments and motor proteins of the myosin II family that coordinately produces tension under the PM of cells. Such PM-associated tension controls cell shape, polarization, motility and cell–cell interactions, among other important cellular functions. In addition to actin and myosin II, the cell cortex contains roughly one hundred actin binding proteins (ABPs) that are involved in organization of the actin meshworks and are important for the generation and regulation of tension near the PM [1]. Ezrin, radixin and moesin proteins (ERMs) and merlin are among the ABPs that regulate organization of actin filaments (F-actin) under the PM (reviewed in [2]). ERMs localize to PM protrusions (e.g., microvilli, filopodia, retraction fibers and pseudopods), cell–cell junctions and the cleavage furrow of dividing cells. The ERM-related protein merlin, the neurofibromatosis type 2 (NF2) tumor suppressor gene product, is also associated with cell–cell junctions. However, its functions in leukocytes will not be addressed in this review since their study has been mainly restricted to cancer cells and cells of the nervous system, in which merlin regulates signaling pathways associated not only with the PM but also with the cytoplasmic and nuclear compartments (reviewed in [3]). Ezrin (named after Ezra Cornell University, where it was isolated) was originally identified as a component of microvilli in chicken intestinal epithelial cells, while radixin (from the Latin radix, which means root) and moesin (membrane-organizing extension spike protein) were isolated from the adherens junctions of rat liver hepatocytes and smooth muscle cells of the bovine uterus, respectively [4,5,6]. By anchoring F-actin to the cytoplasmic tail of transmembrane proteins, ERMs can regulate cortex tension and stiffness throughout the PM and PM-associated domains of polarized cells, taking part in the formation of complex tissue-associated structures including the brush border of intestinal villi [7]; organization of photoreceptors in the retina [8,9]; and formation of tubules in blood vessels, the excretory intracellular canal of *Caenorhabditis*, and terminal cells of the *Drosophila* tracheal system [10,11,12]. Although cultured cells express ERMs to a greater or lesser extent, the expression of particular ERM members is strictly regulated in certain tissues: endothelial cells mainly express moesin, ezrin is expressed in intestinal epithelial cells but is absent in hepatocytes, whereas the opposite holds true for radixin. Moesin is the most abundant ERM in leukocytes, whereas ezrin is less expressed and radixin is nearly absent [13,14,15,16]. In this review, we describe the intrinsic features that enable ERMs to work as efficient PM-cytoskeleton cross-linkers, and offer a perspective on the functional role of ERMs in leukocyte polarization, migration and intercellular adhesion, focusing on the phagocytic cup and the immune synapse (IS) as paradigmatic PM-associated actin-based structures for the function of leukocytes in the immune system.

## 2. ERM Tools for Plasma Membrane-to-Cytoskeleton Bridging

**Figure 1:**
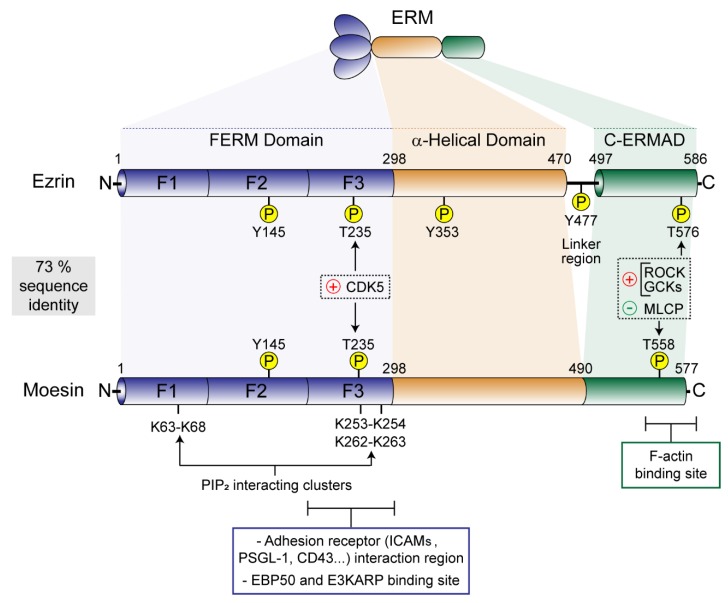
Schematic comparison of the conserved domain structure of human ezrin and moesin showing their sequence identity. The three subdomains (F1–F3) of the N-terminal band 4.1 protein ezrin, radixin and moesin (FERM) domain, the α-helical region, and the C-terminal ERM association domain (C-ERMAD) are depicted. Note that ezrin bears a linker region containing a regulatory Tyr (Y477) that is absent in moesin. The binding sites for PIP_2_, adhesion receptors and the PDZ domain-containing proteins EBP50 and E3KARP in the FERM domain, and for the F-actin binding site in the C-ERMAD, are shown together with the regulatory Tyr and Thr. Ser/Thr-specific ERM-associated kinases (CDK5, cyclin-dependent kinase 5; ROCK, Rho kinase; GCKs, germinal center kinases, e.g., LOK, lymphocyte-oriented kinase) and phosphatases (MLCP, myosin light chain phosphatase) are also depicted.

Given the high degree of homology shared among the three ERMs (73% amino acid identity) and the expression of more than one in many cell types, overlapping or even compensatory functions have been proposed. This suggests that they work in a similar way, a view that has been confirmed at structural level except for some cases in which specific activities have been assigned to individual ERMs. ERMs bear two well-defined functional domains connected through a long α-helix region: the N-terminal FERM (band 4.1 protein-ERM) domain and the C-terminal ERM association domain (C-ERMAD, 50% sequence homology among ezrin, radixin and moesin). The FERM domain is composed of three subdomains (F1, a ubiquitin-like domain; F2, with four α-helices; and F3, a pleckstrin homology domain) and shows over 75% sequence homology [3] (Figure 1). The presence of the FERM domain is critical for the function that ERMs exert as linkers of the PM and the actin cell cortex.

Biochemical studies and structural analyses of protein complexes with the cytoplasmic tail of adhesion molecules ICAM-2, PSGL-1, CD43 and CD44 [17,18,19,20,21] have shown that ERMs can directly bind to these adhesion receptors through a juxtamembrane cytoplasmic region containing a positively charged cluster and a contiguous nonpolar amino acid motif (R/K)-(aa_2_/aa_3_)-(Y/L)-aa-(L/V/I) (where aa represents any amino acid), a finding that can be extended to other known ERM-binding proteins (e.g., ICAM-1 [22], ICAM-3 [23], VCAM-1 [24] and N-CAM-L1 [25]). Such binding to ERMs takes place in a groove formed between a β-strand and an α-helix of the FERM F3 subdomain. In addition to this consensus motif, specific Ser in the cytoplasmic tail of adhesion molecules can regulate their binding to ERMs through phosphorylation-dependent mechanisms. Interactions between Ser and the FERM domain have been reported in ICAM-3, PSGL-1, N-CAM-L1 and L-selectin; whereas phosphomimetic mutations of key Ser residues susceptible to phosphorylation by PKC in the cytoplasmic tail of ICAM-3 (Ser6), CD43 (Ser76), CD44 (Ser2) and L-selectin (Ser9) interfere with their binding to the FERM domain, likely by reducing the net positive charge of their FERM-binding motifs [17,26,27,28,29,30]. The FERM domain can also bind indirectly to ion transporters and other transmembrane receptors (e.g., the β_2_-adrenergic receptor, Na^+^/H^+^ exchangers [NHE3], and the cystic fibrosis transmembrane conductance regulator, CFTR) through two PDZ domains in the scaffolding ERM-binding phosphoprotein 50 (EBP50, also called NHERF1) and NHE3 kinase A regulatory proteins (E3KARP, also called NHERF-2) (reviewed in [31]). Crystal structures of the EBP50 and E3KARP C-terminal peptides bound to radixin have identified a consensus amino acid sequence that can bind to a region of the FERM domain that, despite barely overlapping with the binding site to adhesion molecules, can interfere with their binding by transmitting conformational changes in the F3 subdomain [32].

The FERM domain and the C-ERMAD can bind each other in a head-to-tail manner, leading to a closed/inactive conformation [33,34]. The release of the C-ERMAD from the FERM domain is necessary for the activation of ERMs, unmasking their F-actin- and PM binding sites. The C-ERMAD can also bind to F-actin after phosphorylation on a conserved Thr in ezrin, radixin and moesin (Thr576, Thr564 and Thr558, respectively) [35,36], which is an important feature for the fine regulation of the PM-to-actin cytoskeleton-linking activity of ERMs. Although phosphorylation of Thr in the F-actin binding site containing C-ERMAD is essential for the activation of ERMs, our current view of how ERMs bind to both PM and F-actin requires the participation of phosphatidylinositol 4,5-bisphosphate (PIP_2_) [37,38,39]. Among the mechanisms by which PIP_2_ may regulate activation of ERMs, it is worth noting recent studies suggesting that, during the interaction between CD44 and ERMs, two molecules of ERM and two molecules of CD44 are indirectly bound by PIP_2_ forming a heterotetramer at the PM. PIP_2_ can bind to two sites on the FERM domain (Lys63-Lys68 of the F1 subdomain, and clusters Lys253-Lys254 and Lys262-Lys263 of the subdomain F3) through a mechanism by which one molecule of PIP_2_ sequentially binds the FERM subdomains, changing the conformational structure of ERMs in such a way that renders the F-actin binding site of the C-ERMAD more accessible for Thr phosphorylation [40,41]. Initial studies reported that the key Thr on the C-ERMAD was phosphorylated by Rho-kinase (ROCK) both in vitro and in vivo [36]; however, ROCK-independent mechanisms have also been described, suggesting that C-ERMAD may be phosphorylated by other kinases [42,43]. From then, the number of Ser/Thr kinases that are able to phosphorylate the conserved Thr on the C-ERMAD of ERMs has greatly increased. The PKC isoenzymes PKC-θ and PKC-α phosphorylate moesin and ezrin in vitro and associate with them in human T lymphocytes and breast carcinoma cells, respectively [44,45]. Moreover, recent attention has been given to germinal center kinases (GCK), a subfamily of the mammalian sterile 20-like kinases (Mst) including lymphocyte-oriented kinase (LOK), Mst4, SLK and Nck interacting kinase (NIK), as the main kinases that phosphorylate the regulatory Thr of ERMs during cell motility and division [46,47,48,49,50,51]. To this list of kinases, we must now add two sterile 20-like kinases identified in *Drosophila*, misshapen (an orthologue of NIK) and Slik/SLK [52,53,54].

ERMs are also regulated by phosphatases, as dephosphorylation of the key regulatory Thr of the C-ERMAD detaches ERMs from the cell cortex, adopting a closed/inactive conformation in the cytoplasm. In mammalian cells, several phosphatases can dephosphorylate the regulatory Thr of ERMs. Pioneering studies have reported the association of moesin and ezrin with myosin light chain phosphatase (MLCP) and their coordinated regulation by ROCK-mediated phosphorylation and MLCP-mediated dephosphorylation downstream of the activity of the GTPase Rho [55]. Moreover, protein phosphatase 1 (PP1, the catalytic domain of MLCP) and 2C (PP2C) can dephosphorylate moesin both in vitro and in vivo from human platelets and at the cortex poles of anaphase cells, respectively [56,57]. More recently, involvement of the tumor suppressor PTEN phosphatase in the dephosphorylation of moesin has also been described in chemoattractant-treated neutrophils [58].

Although the regulatory Thr in the C-ERMAD is the most recognized target for phosphorylation-mediated ERM activation, there are other important targets of phosphorylation: Thr235 in the interface between the FERM and the C-ERMAD, the ezrin-specific Tyr353 and 477, and Tyr145 (conserved in all three ERM members) [59,60,61] (Figure 1). Some evidence suggests that, at least in ezrin, Thr235 is phosphorylated by cyclin-dependent kinase 5 (CDK5) and cooperates with Thr576 for its full activation and the cell morphology changes induced in osteosarcoma cells during senescence [62]. On the other hand, ezrin Tyr145, 353 and 477 can be phosphorylated by Src kinases and the intrinsic Tyr kinase activity of the growth factor receptors for EGF, HGF and PDGF. Tyr145 and 477 seem to play a role in cell adhesion and migration, whereas ezrin Tyr353 has been linked to reorganization of the actin cytoskeleton and activation of B cells in response to tetraspanin CD81- and B-cell receptor (BCR)-mediated stimulation [61,63,64,65,66]. However, the importance of these posttranslational modifications on the activation and function of ezrin has been much less studied than the effects of the regulatory Thr of the C-ERMAD. Therefore, although promising, extensive work is required to draw a clear view of the relationship between these posttranslational modifications and how they regulate ERM functions, paying particular attention to the possibility that they may explain some of the specific cellular functions described for ezrin in leukocytes.

## 3. ERMs in Leukocyte Polarization and Migration

In leukocytes, polarization and migration are interconnected processes regulated by ERMs and their interaction with guanine nucleotide exchange factors (GEFs) and Rho GDP-dissociation inhibitors (RhoGDI) of the small Rho GTPases Rho, Rac and Cdc42 in the two major PM-associated compartments of polarized cells, the leading edge and the uropod [67,68]. These two cell poles are characterized by their respective clustering of chemoattractant receptors and enrichment of adhesion molecules on ERM-organized microspikes and microvilli [69,70]. Leukocytes egress from hematopoietic niches and lymphoid organs to patrol the organism following endothelial cell-presented adhesion receptors and chemoattractant trails that permit their exit from blood and lymphatic vessels and arrival to target tissues (e.g., secondary lymphoid organs and inflammatory foci) [71]. Leukocytes responding to chemoattractants convert mechanical forces into directional locomotion as a result of their marked front-to-rear polarity. Hence, Rac-dependent actin polymerization at the leading edge and retraction at the trailing edge by RhoA/ROCK/phosphorylated myosin light chain (MLC)-stimulated actomyosin contraction near the uropod are coordinately regulated by each other to maintain polarity and generate the main forces pushing leukocytes forward [72,73].

**Figure 2:**
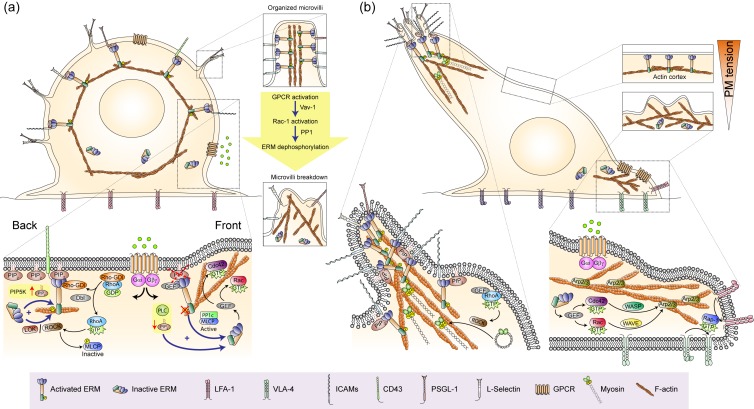
Compartmentalized activation of ERMs and small Rho GTPases in polarized motile leukocytes. (**a**) Initial symmetry breaking events in resting leukocytes stimulated with chemoattractants. In response to chemoattractants, G protein-coupled receptor (GPCR)-associated heterotrimeric proteins stimulate ERM activation at the cell rear through phosphorylation by the kinase activity of LOK and RhoA-stimulated ROCK, production of PIP_2_ by PIP5K; and ERM inactivation at the cell front through PIP_2_ hydrolysis by phospholipase C (PLC) and the phosphatase activity of the PP1 subunit of MLCP, which subsequently activates Rac and Cdc42 through the release of their corresponding guanine nucleotide exchange factors (GEFs). On the upper right, breakdown of microvilli by chemoattractant-stimulated Rac-1 activation is depicted. (**b**) Polarized motile leukocyte showing Rac- and Cdc42-stimulated actin polymerization by the Arp2/3 complex in the leading edge and RhoA-ROCK-mediated actomyosin contraction in the uropod, which provide the main forces required to move the cell forward. Rap-1-mediated β1 (e.g., VLA-4) and β2 (e.g., LFA-1) integrin activation in the leading edge and adhesion molecule-bearing microvilli on the uropod are also depicted. On the upper right, plasma membrane (PM) tension near the uropod and at the leading edge are compared.

Studies with primary T and B lymphocytes, neutrophils and HL-60 human myeloid cells clearly show that a considerable proportion of ERMs are constitutively activated by phosphorylation and bound to the PM of resting leukocytes [16,74,75,76]. PM tension and cell symmetry can be maintained by the inactivation of small Rho GTPases as result of the binding of their corresponding GEFs (e.g., PDZRhoGEF, Vav1 and α-PIX) to activated ERMs. However, cell symmetry can be broken by chemoattractants, which induce leukocyte polarization through transient dephosphorylation of ERMs by the phosphatase activity of the PP1c subunit of MLCP that, in response to the G protein-coupled receptor (GPCR)-associated heterotrimeric protein Gα_i_ and the hematopoietic cell-specific actin regulatory protein Hem-1, is recruited to the emerging leading edge [76]. Once there, MLCP dephosphorylates ERMs, in turn releasing GEFs to activate Rac and Cdc42, stimulating F-actin polymerization and subsequent PM protrusive activity at the cell front. Almost immediately, ERMs can be re-phosphorylated by LOK and/or RhoA-stimulated ROCK and redistributed to the cell rear, reinforcing PM tension and preventing the formation of secondary pseudopods (Figure 2). Adhesion to substratum-coated surfaces (e.g., fibrinogen, fibronectin and VCAM-1) via β1 integrins is also a prerequisite to break the symmetry of leukocytes in chemoattractant-induced polarization. Physical tension induced at the substratum-attached cell rear polarizes SRGAP-2 (a Bin-Amphiphysin-Rvs (BAR) domain containing Rac-1-GAP), which binds and deforms the PM, co-recruiting activated myosin II (pMLC) and the synthesis of PIP_2_ by PIP5K [77,78,79]. Since PIP_2_ is essential for the full activation of ERMs, it is feasible that its synthesis at the rear of adhered leukocytes may contribute to recruitment and retention of ERMs in the emerging uropod. In this regard, there is evidence indicating that ERMs play an important role in the organization of the trailing edge and the formation of the uropod. Phosphorylated ERMs organize at the cell rear with flotillin-containing lipid rafts, forming clusters that can activate RhoA by either sequestering its inhibitor Rho GDI or by binding to Dbl, a Rho GEF concentrated in the cell rear [43,80]. In this cell compartment, activation of Rho kinase (ROCK) by RhoA phosphorylates MLC to stimulate actomyosin contraction, which together with F-actin binding to ERMs and polymerization can form the uropod [43,73]. Thus, in polarized leukocytes, it seems that capping of ERMs at the cell rear not only establishes where the uropod should be formed, but it is actively involved in its formation by activating RhoA-ROCK and maintaining the PM tension required to impede the formation of pseudopods anywhere other than on the leading edge.

Although initial studies did not find any significant differences between the adhesion and migration capabilities of moesin-deficient and wild-type mouse fibroblasts [81], more recent studies have suggested a non-redundant role for ezrin and moesin in lymphocyte migration. The number of lymphocytes that egressed from primary and secondary lymphoid organs was impaired in the moesin-knockout mice, reducing the populations of T and B cells in the peripheral blood and lymph nodes [82]. Furthermore, siRNA silencing of moesin in T lymphocytes from conditional ezrin-deficient mice showed that ERMs are important for integrin β1-mediated adhesion to fibronectin and homing to lymphoid organs, but for chemotaxis solely when cells must pass through constricted spaces [79]. In this regard, an increasing body of evidence suggests that ERMs are involved in both intra-tissue leukocyte chemotaxis and in the main steps of the chemoattractant-stimulated leukocyte recruitment cascade: tethering, rolling, firm adhesion and transendothelial migration (TEM) [83].

### 3.1. Tethering and Rolling

Activated ERMs and ERM-binding adhesion receptors (e.g., PSGL-1, L-selectin, ICAMs, CD43 and CD44) work together in the organization of microvilli, F-actin-based finger-shaped PM protrusions that are important for tethering and rolling during the initial contacts of leukocytes with endothelial cells [84,85]. It has been proposed that in the bloodstream, PSGL-1 and L-selectin on the tips of leukocyte microvilli are the first adhesion molecules that establish contact with their counter-receptors (E/P-selectin and CD34, respectively) on activated endothelial cells of postcapillary venules. L-selectin also contacts the addressins MadCAM-1 and GlyCAM-1 on the high endothelial venules (HEVs) of lymphatics, or even with PSGL-1 during secondary contacts among bystander and adhered leukocytes. In this regard, there is much evidence indicating that moesin and ezrin regulate the tethering and rolling velocity of leukocytes both in vivo and in vitro. Deficiency of Rap-1, a small Rho GTPase that in its inactive form fosters ERM phosphorylation in resting leukocytes by activating LOK, disturbs rolling of naïve T lymphocytes on P-selectin and on addressins by inhibiting tethering [86]. The rolling velocity of neutrophils in cremaster muscle venules after trauma- or TNF-α-induced inflammation is detrimentally higher in moesin-deficient than control mice [87]. 32D myeloblast-like cells expressing an ERM-binding defective PSGL-1 mutant show increased rolling velocity and reduced tethering on L-, P- and E-selectin, whereas only tethering on PSGL-1 is affected in the corresponding ERM-binding defective L-selectin mutant [85,88]. Lastly, treatment of mouse splenic B lymphocytes with the phosphatase inhibitor calyculin A (which increases ERM phosphorylation) or overexpression of a phosphomimetic ezrin mutant impairs microvilli formation, chemotaxis and B cell migration to the spleen and lymph nodes [14]. This suggests that ERMs may support early steps in the leukocyte transmigration cascade, including secondary leukocyte tethering on leukocytes adhered to endothelial cells, through the formation of PSGL-1- and L-selectin-bearing microvilli.

### 3.2. Firm Adhesion

Rolling leukocytes can detect chemoattractants bound to proteoglycans on endothelial cells and spread on them upon binding to G protein-coupled receptors (GPCRs), which can trigger breakdown of microvilli through the transient dephosphorylation of ERMs by Rac-1- and Rap-1-mediated PP2A/PP1 activation and LOK inhibition, respectively [86,89]. This early morphological alteration may help cells to reduce PM tension and increase the surface of contact at the leukocyte–endothelial cell interface. In addition, chemoattractants induce clustering and conformational activation of the β1 integrin VLA-4 and β2 integrins LFA-1 (in lymphocytes and monocytes) and Mac-1 (in monocytes and neutrophils) and their respective interaction with VCAM-1 and ICAM-1. The latter are organized in the apically localized tetraspanin microdomains of endothelial cells, adhesion platforms that can be linked to the actin cytoskeleton through the binding of CD81 and its partners EWI-2 and EWI-F to ERMs [90,91], giving rise to leukocyte crawling and subsequent firm adhesion and arrest. However, endothelial cells also participate actively in this process. In response to the interaction of β2 integrins with ICAM-1, they form an F-actin-based docking structure which embraces the leukocyte with ICAM-1- and VCAM-1-rich PM projections containing PIP_2_ and phosphorylated moesin and ezrin (among other ABPs) [24]. This actin cytoskeleton-integrated structure is thought to enable the dynamic transition between leukocyte firm adhesion and TEM. Although the interaction between VCAM-1 and moesin is involved in leukocyte adhesion to the endothelium, PM projections containing moesin and ICAM-1 facilitate TEM [24,92]. The contribution of ERMs to the formation of the docking structure and their role in leukocyte adhesion and TEM have been studied both in an experimental model of leukocyte chemotaxis in COS-7 cells expressing an ERM-binding defective mutant of ICAM-1, and also in human endothelial cells infected with *Neisseria meningitidis*, in which the pathogen competes with leukocytes for the recruitment of ERMs, ICAM-1 and VCAM-1 to their adhesion sites. Overexpression of ezrin and moesin in *N*. *meningitidis*-infected endothelium rescued the formation of the docking structure, whereas expression of the ezrin FERM domain blocked its formation along with adhesion and diapedesis of leukocytes, similarly to the effect of expression of the ERM-binding defective ICAM-1 mutant in COS-7 cells [93,94], confirming that the complex organization of adhesion receptors and F-actin in the docking structure is regulated by ERMs.

### 3.3. Transendothelial Migration

To cross the endothelial cell monolayer and reach the abluminal side of the vessel, leukocytes must pass through narrowed spaces, overcoming the resistance offered by endothelial cells and their basement membrane. To achieve this, PM deformation, force-generated extension at the leading edge and retraction of the uropod are required [95]. Chemoattractant-induced ERM dephosphorylation regulates PM tension and deformation in neutrophils and T lymphocytes. The importance of the regulation of PM tension by ERMs during TEM has been studied in transgenic mice constitutively expressing the phosphomimetic ezrin mutant T567E, whose T lymphocytes present increased PM tension, defective migration in vitro and impaired homing to lymph nodes [96]. Although these lymphocytes showed no defective tethering and rolling on HEVs (perhaps because, as noticed by the authors, these cells did not present alterations in the length and number of microvilli, possibly as a consequence of the low levels of ezrin T567E expressed in cells), their passage to the parenchyma was seriously impaired, as most of them either did not cross the endothelial monolayer or took much longer to cross it than wild type lymphocytes. Accordingly, neutrophils from PTEN knockout mice, a phosphatase that can dephosphorylate moesin on Thr558, are defective in chemotaxis and recruitment to the peritoneum in a thioglycolate-induced mouse model of acute peritonitis [58]. Furthermore, treatment of T lymphocytes with glucocorticoids, which increase ERM phosphorylation through gene expression-independent mechanisms, reduce transmigration in vitro [97]. On the other hand, leukocytes crawling on endothelial cells under shear stress can ventrally extend short, Cdc42-dependent exploratory filopodia and Wiskott–Aldrich syndrome protein (WASP)-regulated invasive podosomes for transcellular and paracellular TEM, respectively. These are reorganized into a main pseudopod when an appropriate place for diapedesis, the passage of leukocytes through capillary endothelial cells, is found [98,99]. WASP is considered an effector of many of the mechanisms by which Cdc42 promotes actin polymerization in leukocytes. Further studies have shown that a deficiency of Cdc42 interacting protein 4 (CIP4), which interacts with both Cdc42 and WASP, impairs interaction of T lymphocytes with immobilized ICAM-1 and VCAM-1 under shear flow and their transmigration across TNF-α-activated endothelial cells [100]. Knowing whether regulation of ERM dephosphorylation can facilitate this passage by fostering PM deformation and activating Cdc42-dependent actin polymerization for formation of the invasive pseudopod would thus provide new insights on how TEM is regulated. Interestingly, recent findings from the study of inflammatory monocytes suggest that ERMs can work differentially in TEM, since ezrin binds first to L-selectin and promotes formation of a main pseudopod, whereas moesin interacts later on, fostering shedding of the L-selectin ectodomain and restricting the appearance of additional pseudopods that would disturb directional transmigration [30,101].

On the other hand, taking advantage of the fact that moesin is the sole ERM member expressed in *Drosophila*, an important role for moesin in control of the leading pseudopod has been also associated with persistence and directionality of collective cell migration in vivo, a process by which groups of cells coordinately move through tissues [102]. In border cells of the egg chamber in the *Drosophila* ovary, which form a small cluster that migrates directionally by means of a pseudopod strictly formed at the front of the cluster’s leader cell, silencing of moesin or the moesin kinase misshapen promotes formation of protrusions in non-leader cells and disturbs polarized migration of cell clusters [52,103], suggesting that localization of moesin at cell–cell contacts can foster formation of the leading pseudopod by increasing cortical membrane stiffness. In the immune system, most leukocytes move as solitary cells, but in certain lymphoid malignancies they can also move as aggregates in tissues [104]. This opens the interesting question of whether ERMs may also regulate the collective migration of leukocytes by preventing formation of additional pseudopods in non-leader cells.

## 4. ERMs and Intercellular Adhesion: the Phagocytic Cup and the Immune Synapse as Paradigms of Ezrin- and Moesin-Mediated PM Organization in Leukocytes

In order to exert cytokine secretion-independent defensive functions, macrophages, DCs, neutrophils and other phagocytic leukocytes must establish contact with microorganisms, tumoral and apoptotic cells in a pathophysiological context using PM-associated receptors (e.g., high-affinity IgG-binding receptors (FcγRs), complement receptor (CR)3, toll-like receptor (TLR)-4, the receptor of apoptotic cells stabilin-2, or the scavenger receptors CD36 and Dectin-1). This contact permits their engulfment via the phagocytic cup and internalization within the phagosome, an intracellular vesicle formed by invagination of the PM. The phagosome is responsible for degradation and, in the case of professional antigen-presenting cells (APCs), ultimate processing of proteins to initiate adaptive immune responses by presenting antigen to the antigen-specific T cell receptor (TCR) at the immune synapse (IS), a PM-associated intercellular compartment that regulates the activation of T lymphocytes by APCs, but that is also found in NK cells and B lymphocytes [105,106,107].

**Figure 3:**
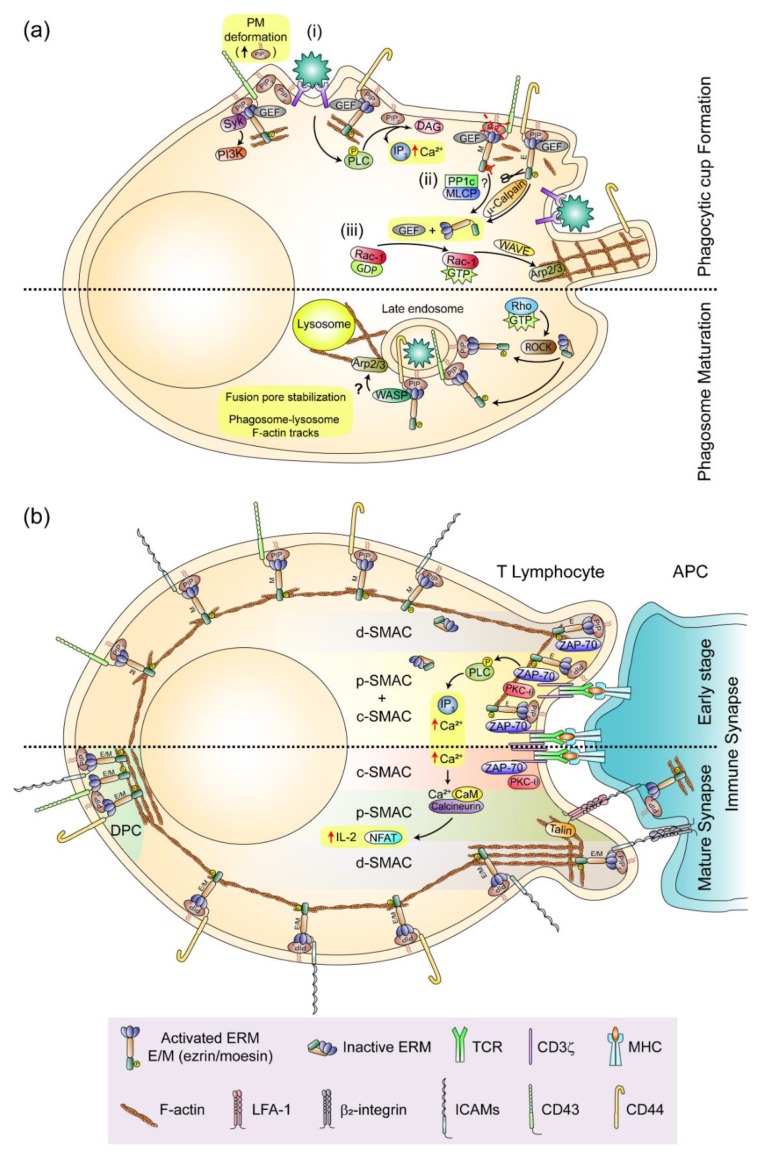
Organization of leukocyte interactions by ERMs. (**a**) The phagocytic cup and the phagosome. Upper part, three-step phagocytic cup formation in direct receptor-mediated phagocytosis (e.g., scavenger and apoptotic cell recognition receptors): (i) receptor-mediated cell binding and recruitment of PIP_2_ and ERMs to deformed PM. PLC and Syk signaling from the phagocytic receptor and the ERM-bearing immunoreceptor tyrosine-based activation motif (ITAM) are respectively depicted; (ii) activation of PLC from the phagocytic receptor reduces PM PIP_2_ and increases intracellular Ca^2+^ levels, which may release ERMs from the PM and activate Rac-1 through the specific cleavage of ezrin by calpain and dephosphorylation of ERMs by myosin light chain phosphatase (MLCP); (iii) activation of Rac-1 by GEFs released from inactivated/closed ERMs may stimulate actin polymerization by the WAVE-Arp2/3 complex, giving rise to the phagocytic cup. Lower part, phagosome maturation: In late phagosomes, ERMs return to their intracellular side through a Rho-dependent mechanism, promoting phagolysosome formation through WASP-ARP2/3-mediated actin polymerization. (**b**) The T cell immune synapse. Upper part, early immune synapse (IS) formation: ERMs are transiently inactivated and ezrin interacts with ZAP-70 along the IS fostering intracellular fluxes of Ca^2+^, whereas moesin and the adhesion molecules CD43, CD44 and ICAMs are excluded from the IS. Lower part, mature IS: Ezrin and moesin are both excluded from the IS and preferentially localized together with adhesion molecules and F-actin at the d-SMAC and the distal pole complex (DPC), promoting the activation of TCR-associated proximal and distal elements (PLC-γ activation/Ca^2+^ mobilization and NFAT dephosphorylation/IL-2 production, respectively).

Although the phagocytic cup and IS are quite different structures in regard to the identity of the PM-associated receptors and cell types involved in their formation, they still share some important features [108]. Among them, it is worth noting the involvement of the actin cytoskeleton and ERMs in the dynamics of receptors driving their functional organization (Figure 3).

### 4.1. The phagocytic Cup and the Phagosome

ERMs and small Rho-GTPases are involved in the early and late steps of phagosome formation. Phagocytic leukocytes expand their PM surface upon receptor-mediated contact with target cells, forming the phagocytic cup. ERMs control lateral diffusion of receptors on PM by forming a fence-and-picket structure (also termed “corral”) of F-actin bundles indirectly bound to transmembrane proteins (e.g., CD44) through ERMs [109]. This constraint is less strict in the leading edge of phagocytic leukocytes, where receptors can diffuse early upon ligand recognition to form clusters that bind multivalent targets, and may be subsequently confined at the emerging phagocytic cup by ERM-sustained corrals. However, considering that ERMs can also work as protein-associated modules to transmit signaling through the binding of the Src kinase Syk to the immunoreceptor tyrosine-based activation motif (ITAM) of their FERM domain [110], a provocative study has suggested that ERMs may stimulate phagocytosis through receptor-independent mechanisms. In this way, they would act as phylogenetically conserved mechanotransducers that activate PI3K in response to the deformations of the PM, which accumulate PIP_2_ at the contact sites with foreign cells and particles, recruiting ERMs to the emerging phagocytic cup [111]. Nevertheless, while ERMs can regulate phagocytosis through a signaling pathway similar to that triggered by the ITAMs of FcγRs, the absence of moesin does not disturb internalization of IgG-coated particles, suggesting that ERMs are not essential for opsonization-mediated internalization. On the contrary, ERMs seem to improve phagocytosis mediated by those mechanisms in which neither opsonization nor ITAM-bearing receptors are involved, such as the clearance of microorganisms and apoptotic cells by direct binding of scavenger receptors and receptors of phosphatidyl serine (PS), respectively [111]. Although attractive, the mechanotransduction model by which the PIP_2_–ERM–ITAM–Syk axis may collaborate with receptor-mediated opsonisation-independent phagocytosis leads to some unanswered questions. For instance, since ezrin and moesin are already activated at the PM of resting leukocytes, connecting the PM to the actin cortex in flat areas as well as in microvilli and other small protrusions on which the phagocytic cup will be formed, the positioning of ERMs at the PM seems not to be sufficient for phagocytic cup formation, suggesting that additional factors must cooperate with them to trigger phagocytosis. Moreover, some evidence suggests that ERMs detach from the PM early during the phagocytic process, only returning once the phagosome has been formed [112]. In this regard, a specific role for ezrin in rearrangements of the actin cytoskeleton leading to the growth of PM projections surrounding the phagocytic cup of neutrophils has been proposed on the basis that ezrin is the sole ERM regulated by the proteolytic activity of µ-calpain [113]. Ligand binding to phagocytic receptors activates phospholipase (PL)C/D to produce IP_3_, which can trigger an increase of intracellular Ca^2+^, inducing the translocation of µ-calpain from the cytosol to the emerging phagocytic cup where it is activated to cleave ezrin and break the linkage between the PM and the actin cortex (reviewed in [114]). The specific role of ezrin in the formation of the phagocytic cup is supported by a recent report showing that ezrin (but not moesin) promotes the formation of functional phagocytic cup-like invasive structures during infection of mammalian cells by extracellular amastigotes of *Trypanosoma cruzi* [115]. Detachment of ezrin from the PM may relax it but also release Rac-1-GEFs and thereby stimulate activation of Rac-1 and its effector WASP-family verprolin-homologous protein (WAVE), which can trigger localized actin polymerization by the Arp2/3 complex, pushing the PM away to form the phagocytic cup [116]. On the other hand, in using FRET-based biosensors to visualize the activity of small Rho-GTPase in real-time imaging, recent studies on the clearance of apoptotic thymocytes through stabilin-2, one of the PS receptors, have shown that RhoA is transiently activated immediately before phagocytic cup closure and internalization. Furthermore, a constitutively activated form of RhoA inhibits phagocytosis through ROCK, suggesting that besides Rac-1, RhoA is also important for early phagocytic events [117]. In macrophages, ROCK regulates maturation of phagosomes generated from the clearance of apoptotic cells [118], a function that would take place through the re-phosphorylation of ERMs and their binding to PIP_2_ and/or some of their abundantly expressed PM partners (e.g., CD43 and CD44) on the cytoplasmic side of late phagosomes. At this site, ERMs can interact with WASP via the FERM domain, and induce de novo F-actin polymerization through Arp2/3, enabling phagolysosome formation by transmission of lysosomal content to late phagosomes [112] (Figure 3a). Nevertheless, how ERMs can facilitate phagosome–lysosome fusion is still an open question. Although some authors assign a direct action to ERMs in the stabilization of the fusion pores that connect the two organelles during the acidification of the phagosome [119], the possibility that localization of ERMs on phagosomes may still provide F-actin tracks for phagosome–lysosome approach and membrane fusion seems not be entirely excluded.

### 4.2. The Immune Synapse

To carry out their functions during adaptive immunity, lymphocytes must first be activated in the IS. The prototypical IS of T cells is organized in a central area or supramolecular activation cluster (c-SMAC) in which the TCR-CD3 signaling complex is concentrated, a ring-shaped peripheral (p)-SMAC where the β2 integrin LFA-1 is clustered with talin and β-actin, and a more distal lamellipodium-like (d)-SMAC characterized by a ring of F-actin [120]. Among its functions, the IS modulates T cell activation, attenuating or sustaining signaling by degradation of TCR-ligand complexes at the c-SMAC or stabilization of signaling microclusters at the p-SMAC, respectively [121,122].

Several research groups simultaneously reported the localization of ezrin and moesin at the T cell IS, and they explored their possible functions in its organization with a focus on their phosphorylation and co-distribution with the large ERM-binding glycoprotein CD43 (Figure 3b). They found that ERMs were transiently dephosphorylated in response to TCR-triggered stimuli and localized with F-actin at the d-SMAC, or beyond it, in the distal pole complex (DPC) when T cells adopt a striking polarized morphology, excluding CD43 from the IS [123,124,125]. This ERM function was also observed in the killer cell immunoglobulin-like receptor (KIR)- and NKG2A receptor-triggered inhibitory IS of NK cells, but only partially in the NK cell activating IS in which ezrin co-localizes with granules of perforin, whereas moesin can be redistributed towards the DPC [15,126,127]. While exclusion of CD43 from the IS was initially regarded as a mechanism to facilitate T cell activation by preventing steric interference of TCR binding to major histocompatibility complex (MHC)-loaded antigenic peptides, further studies with hyperproliferative CD43-deficient T cells expressing CD43 protein constructs have shown that regulation of T cell activation by CD43 is not exerted by its extracellular domain, but rather by signaling from its cytoplasmic tail independently of where it was localized on the PM [128]. Later, interesting nonredundant roles for moesin and ezrin in organization and function of the IS of T cells were proposed in light of results showing that ezrin was specifically localized in the IS associated with ZAP-70, a key adaptor-kinase for TCR-triggered signal transmission to proximal elements of activation (e.g., the adaptor protein linker for activation of T cells LAT), whereas moesin excludes CD43 from the IS [129]. Moreover, by expressing phosphomimetic- and phosphorylation-defective ERM mutants in Jurkat cells, the study showed that phosphorylation of ezrin on Thr567 and moesin on Thr558 are both important for IS formation, but that only phosphorylation of ezrin was involved in the recruitment of ZAP-70 to the IS and TCR-triggered Ca^2+^ mobilization. However, in using T lymphocytes from ezrin-knockout mice, another study has shown that ezrin can exert a slight impact on the organization of the IS, and although ezrin (but not moesin) is recruited to the IS at early times, both are subsequently recruited to the DPC, working together to promote activation of TCR proximal (PLC-γ and ZAP-70 phosphorylation; and Ca^2+^ intracellular flux) and distal (NF-AT transcriptional activity and IL-2 production) elements [130]. The apparent discrepancies between these two studies have been mainly assigned to differences in the cellular models used (human Jurkat T cells vs. primary mouse T lymphocytes) [131], off-target effects of the exogenously expressed ERM mutants, possible cross-reactivity of the Abs used, and residual endogenous expression of moesin in ezrin knockout mouse T cells interfered with moesin-specific siRNAs. Regardless, there seems little doubt that ERMs are involved in organization of the IS, a role also supported by the finding that efficient formation of antigen-specific T cell–APC conjugates requires Vav1-Rac-1-regulated ERM dephosphorylation and subsequent PM detachment from the actin cortex at the T cell–APC contact interface [132]. This may promote conjugate formation by increasing PM flexibility and the avidity of LFA-1 for ICAM-1 on the APC, since the transient detachment of LFA-1 from depolymerized F-actin can increase its lateral mobility to form small aggregates and clusters at the p-SMAC [133]. However, ERMs can also regulate the interaction between LFA-1 and ICAM-1 from the APC side of the IS. Anchoring of ICAM-1 to the actin cortex can provide the resistance required to stretch the F-actin-associated β2 chain of LFA-1, thus inducing the conformational changes required to increase the affinity of LFA-1 for ICAM-1 [134]. This function is also observed in the activating IS of NK cells, in which ERMs tether ICAM-1 to the actin cortex of NK-sensitive target cells, facilitating the polarized secretion of cytolytic granules through the interaction between ICAM-1 and LFA-1 [135].

ERMs have been also involved in the formation of the B cell IS. In resting B lymphocytes, BCRs are included in lipid rafts, and their diffusion is constrained by a ERM-sustained F-actin corral [136,137]. However, like in TCR-stimulated T cells, ERMs are transiently dephosphorylated in B lymphocytes upon BCR-mediated stimulation, inducing actin depolymerization and the dissociation of PM-associated BCR-containing lipid rafts from the actin cortex, which facilitates their coalescence at the IS [138]. BCR triggering induces B cell spread on APCs, giving rise to the rapid ERM-mediated disorganization of the corral’s F-actin, which can permit signaling by lateral diffusion of small, BCR-containing microclusters to form larger, more stable ones that, after ERM re-phosphorylation, re-attach to the PM and are redistributed towards the c-SMAC for internalization by the combined actions of the centripetal retrograde flow of actin and actomyosin contraction (reviewed in [137]). The importance of ERMs for organization of signaling microclusters at the IS of B cells has been demonstrated by overexpression of the FERM domain of ezrin, which disturbed the coalescence of BCR microclusters to the c-SMAC, similar to that previously described for TCR microclusters in the T cell IS [123]. Furthermore, overexpression of the phosphomimetic ezrin T567D mutant or a lack of both ezrin and moesin also impaired the coalescence of BCR microclusters to the c-SMAC [75], suggesting that coordinated ERM binding to and detachment from the PM and F-actin are required for actin cytoskeleton-regulated BCR signaling from microclusters. More recently, a differential function for ERMs has been established between naïve and germinal center (GC) B cells, since the latter form ezrin and moesin-containing podosome-like projections with BCR clusters at their tips, which facilitate antigen extraction from the PM of APCs through “pulling” forces that preferentially engage high-affinity antigens, perhaps to establish thresholds for antigen processing that would permit the spatial-temporal regulation of antigen presentation to follicular T cells [139].

## 5. ERMs and Immune Regulation

A large body of evidence indicates that ERMs are important for the function of lymphocytes. X-linked moesin-associated immune deficiency (X-MAID), a human genetic disorder caused by the missense mutation R171W in the moesin gene, is characterized by extensive lymphopenia, resembling the phenotype observed in moesin-knockout mice, with low proliferation of T lymphocyte in which the proportion of naïve CD4^+^ and senescent and exhausted CD8^+^ T cell subtypes is unusually high [82,140,141]. On the other hand, overexpression of a phosphomimetic moesin mutant attenuates spontaneous autoimmunity in Rap-1-deficient mice by reducing the number of inflammatory T lymphocytes recruited to the colon, whereas T lymphocytes infiltrating inflamed kidneys from systemic lupus erythematosus (SLE) patients show high levels of ERM phosphorylation [86,142]. However, ERMs do not only regulate autoimmunity through cell migration but also by promoting the production of CD4^+^ and CD8^+^ regulatory T cells (Tregs). CD4^+^ Treg production is stimulated by ERM binding and stabilization of the TGF-βR-I and -II on the PM as a positive TGF-β-dependent feedback mechanism that increases the expression of moesin in cells, whereas CD8^+^ Tregs are incremented by fostering the IL-15 signaling pathway that maintains their homeostasis [143,144]. Moreover, ezrin also regulates B cell-mediated autoimmunity, since conditional deletion of ezrin attenuates lupus in mice deficient for the Src kinase Lyn by reducing B cell activation, leukocyte infiltration and IgG deposition in the kidney glomeruli [145]. Regardless, no important defects in the homeostasis of B lymphocytes have been observed in ezrin-deficient mice except for an increase in IL-10-producing Bregs, which make conditional ezrin-deficient mice more prone to trigger pro-inflammatory responses to sublethal doses of LPS in vivo by increasing IL-10 production via TLR4, an effect also observed in B cells treated with an ezrin-specific inhibitor that dephosphorylates ezrin on Thr567 [146,147]. Altogether, these reports suggest that the precise threshold of ERM phosphorylation on the regulatory Thr of the C-ERMAD is critical to prevent defective lymphocyte functions.

## 6. Conclusions and Future Perspectives

A large body of evidence now indicates that ERMs are multifunctional proteins that, through complex regulation by kinases and phosphatases, organize the PM and actin cortex and transmit information between the external milieu and the cell in a bidirectional way, thus linking PM structure to function. In leukocytes this feature is paradigmatic, since ERMs contribute to the process of leukocyte polarization and migration through the control of PM tension and the formation of uropods and microvilli, and they regulate intracellular signaling in lymphocyte activation by locally organizing signaling receptors at the IS.

Although it has been well established that many ERM functions are exerted through activation of small Rho GTPases upon closing/inactivation, it remains unclear how RhoA is activated in the uropods of migrating leukocytes while ERMs can maintain a GEF-sequestering open/active conformation at the PM. In this regard, the mechanism by which ERMs and their partners are relocated to the cell rear during leukocyte polarization (the uropod of motile leukocytes and the DPC of the IS) is still not well understood. Whether the redistribution of ERM-adhesion receptor complexes towards the cell rear is the result of centripetal retrograde flow of the actin cortex, consecutive cycles of ERM phosphorylation/re-phosphorylation, or simply the reassembly of ERMs and their adhesion receptor partners at the actin cortex of the cell rear, remains to be investigated in depth.

Another open question is whether microtubules (MTs), whose centrosome-organized network is packed in the uropod of polarized motile leukocytes, play any role in the function of ERMs. Although pioneering studies on the organization of the erythrocyte marginal band and the association of ezrin with cytoskeletal components in insect cells have suggested some interaction between ERMs and MTs [148,149], it was not until recent studies, which showed an important role for MTs in the mechanisms by which Dmoesin controls the stability and orientation of microtubules in the mitotic spindle of *Drosophila* cells, that the interaction between ERMs and MTs at the cell cortex was reported [53,150]. In this regard, some findings support the notion that ERMs can also interact with MTs in leukocytes. MT disruption by nocodazole breaks polarity and induces clustering of ERMs in T lymphocytes, probably through the release of a Rho-GEF from MTs and the subsequent activation of RhoA-ROCK [151]. Also, the persistence of directional migration of neutrophil-like cells toward chemoattractant gradients has been proposed to be dependent on the polarization of ERMs at the cell rear and their regulation by MTs, since their disruption reduced persistent directional migration through moesin mislocalization [152]. Moreover, ezrin can regulate the organization and function of TCR microclusters in the T cell IS by binding to the PDZ domain containing scaffold protein Dlg1, which may facilitate the interaction of MTs with the cell cortex and, therefore, the organization and function of the MT network in the IS [153].

Further studies will be required to explore the significance of the possible interaction between ERMs and MTs in leukocytes and the impact that ERMs might exert on their function as pivotal linkers of the PM to both the actin and tubulin cytoskeletons.
